# SpeckleNN: a unified embedding for real-time speckle pattern classification in X-ray single-particle imaging with limited labeled examples

**DOI:** 10.1107/S2052252523006115

**Published:** 2023-07-18

**Authors:** Cong Wang, Eric Florin, Hsing-Yin Chang, Jana Thayer, Chun Hong Yoon

**Affiliations:** a SLAC National Accelerator Laboratory, 2575 Sand Hill Road, Menlo Park, CA 94025, USA; Brookhaven National Laboratory, USA

**Keywords:** SpeckleNN, coherent X-ray diffractive imaging, CXDI, X-ray free-electron lasers, single-particle imaging, viruses, speckle patterns

## Abstract

With X-ray free-electron lasers (XFELs), it is possible to determine the three-dimensional structure of noncrystalline nanoscale particles using X-ray single-particle imaging (SPI) techniques at room temperature. Classifying SPI scattering patterns, or ‘speckles’, to extract single-hits that are needed for real-time vetoing and three-dimensional reconstruction poses a challenge for high-data-rate facilities like the European XFEL and LCLS-II-HE. Here, we introduce SpeckleNN, a unified embedding model for real-time speckle pattern classification with limited labeled examples that can scale linearly with dataset size. Trained with twin neural networks, SpeckleNN maps speckle patterns to a unified embedding vector space, where similarity is measured by Euclidean distance. We highlight its few-shot classification capability on new never-seen samples and its robust performance despite having only tens of labels per classification category even in the presence of substantial missing detector areas. Without the need for excessive manual labeling or even a full detector image, our classification method offers a great solution for real-time high-throughput SPI experiments.

## Abstract 2

This paper presents a neural network model called SpeckleNN for real-time classification of X-ray single-particle imaging (SPI) speckle patterns, ideal for use in high-data-rate facilities like the European XFEL and LCLS-II-HE. It possesses the capability to carry out few-shot classification on novel samples and demonstrates robust performance even in the presence of substantial missing detector area, rendering it an excellent candidate for real-time high-throughput SPI experiments.

## 1. Introduction

Single-particle imaging (SPI) with X-ray free-electron lasers (XFELs) is a promising method for determining the three-dimensional structure of noncrystalline nanoscale particles at room temperature. In SPI experiments, intense femtosecond coherent X-ray beams strike biomolecules injected into the beam path, causing radiation-damage-free scattering of the samples. This method of collecting scattering datasets is known as ‘diffraction before destruction’ (Neutze *et al.*, 2000 ▸; Chapman *et al.*, 2006 ▸; Seibert *et al.*, 2011 ▸; Aquila *et al.*, 2015 ▸; Reddy *et al.*, 2017 ▸). Such scattering patterns are also referred to as ‘speckles’ due to their grainy appearance. A single particle of interest can then be reconstructed by algorithms, such as EMC (Loh & Elser, 2009 ▸; Ayyer *et al.*, 2016 ▸) and M-TIP (Donatelli *et al.*, 2017 ▸; Chang *et al.*, 2021 ▸), from hundreds to tens of thousands of speckle patterns.

In today’s SPI experiments, speckle patterns form in four main categories, depending on what interacts with the X-ray pulse at the point of interaction. A large fraction of X-ray pulses may miss the target particle, for example Shi *et al.* (2019 ▸) reported 98% of the pulses did not interact with the sample, resulting in no scattering pattern, defined as a ‘no-hit’. In contrast, a speckle pattern is labeled as a single-hit when X-ray photons collide with one and only one sample particle. Similarly, a multi-hit happens when an X-ray pulse intersects with two or more sample particles. In some cases, X-ray pulses might also hit objects that are not the sample of interest in the delivery medium, and those speckle patterns are defined as ‘non-sample-hit’. The main goal of this work is to provide an efficient solution to identify single-hit speckle patterns in near real time during data collection.

Real-time speckle pattern classification for SPI experiments is a challenge faced by high-data-rate facilities like the European XFEL and LCLS-II-HE, due to their need (1) for real-time vetoing to better utilize data storage and (2) to enable near real-time feedback of reconstructed electron densities. The classification algorithm needs to scale linearly to handle the vast amount of data they generate in real time. Some pioneering works addressing the challenge employed unsupervised learning techniques (Yoon *et al.*, 2011 ▸; Giannakis *et al.*, 2012 ▸; Schwander *et al.*, 2012 ▸; Yoon, 2012 ▸; Andreasson *et al.*, 2014 ▸; Bobkov *et al.*, 2015 ▸). Such solutions can reveal underlying clusters of data categories and runs without human labeling, but require post-human interpretation to achieve reasonable classification results, such as specifying the decision boundary of single-hit speckle patterns in some vector space. Also, these algorithms do not scale linearly with the number of speckle patterns needed for real-time classification. On the other hand, supervised learning solutions based on artificial neural network (NN) models (Shi *et al.*, 2019 ▸; Ignatenko *et al.*, 2021 ▸) scale linearly, but require hundreds of labeled examples of the data being collected during beam time as well as additional time for model training which precludes real-time classification. Such models are made of largely two components: (1) convolutional neural networks (CNNs) for spatial feature extraction and (2) fully connected networks (FCN) for compressing CNN features into a probability distribution of possible outcomes. These models demonstrate good performance on speckle patterns of one single-particle sample, bacteriophage PR772, which is an important step towards the goal of near real-time particle classification. But when studying a different sample, they will have to be retrained on hundreds of labeled speckle patterns. Notably, it is not a lack of computing power that prevents these models from working in near real time, as deep-learning models can be run on supercomputers with modern graphics processing units (GPUs). The bottleneck is speckle pattern labeling. It is by no means a trivial task to label speckle patterns, especially at scale, even for experts in the field. Therefore, we need solutions that enable NN models to effectively classify speckle patterns without excessive manual labeling.

We aim to address the problem of near real-time speckle pattern classification by converting it into the task of measuring speckle pattern similarities. To accomplish this goal, we propose to train an NN model to learn an embedding function, capable of mapping speckle patterns into a unified embedding vector space. An important property of this vector space is that similarities can be evaluated by computing the Euclidean distance between any two points in the space. Then, we classify unknown examples by comparing them with a few labeled examples per class in the embedding vector space and assigning the label of the closest class.

A contrastive approach, known as twin NNs, is used for training the unified embedding model. The main idea is that two identical networks will extract features from a pair of examples, either with the same label or different labels. For two examples with the same label, their Euclidean distance should be small and vice versa. Contrastive approaches based on twin NNs achieved early success in computer vision tasks such as signature verification (Bromley *et al.*, 1993 ▸) and face verification (Chopra *et al.*, 2005 ▸; Schroff *et al.*, 2015 ▸). Moreover, two twin NNs can work together to collectively train the underlying embedding network by minimizing the Euclidean distance between identically labeled examples, while simultaneously maximizing the Euclidean distance between differently labeled examples. This approach is also referred to as triplet networks detailed in the face verification model (Schroff *et al.*, 2015 ▸).

In this work, we present SpeckleNN as a unified embedding network for classifying speckle patterns in real-time X-ray SPI. Specifically, two classification solutions are proposed, one with offline training and one with online training.

First, we show that SpeckleNN accurately classifies speckle patterns with only a few labeled examples per category (*e.g.* 5 examples per category) by learning a unified embedding function from a number of distinct protein samples. The model can be trained entirely offline or prior to data collection, and its classification capability generalizes to new samples.

Second, we demonstrate that SpeckleNN achieves accurate and robust speckle pattern classification in the presence of missing detector area (*e.g.* 25% of a pnCCD detector). The model is designed to be trained online or during data collection on a relatively small number of labeled examples per category (*e.g.* a total of 60 patterns with 40 for training and 20 for validation). Unlike the offline solution, the online training utilizes only the speckle patterns from the sample of interest as opposed to generalized embedding of multiple samples.

## 2. Related work

### 2.1. Speckle pattern classification

The task of single-particle speckle pattern classification often requires expert knowledge and immense manual effort. A human-engineered feature extractor and unsupervised learning came along to tackle this challenge. For instance, spectral clustering (Yoon *et al.*, 2011 ▸), principal component analysis (PCA) and support vector machines (SVMs) (Bobkov *et al.*, 2015 ▸) were employed for single-hit classification. Geometric machine learning is a supervised learning solution based on the diffusion map framework that can output a score for how likely a speckle pattern is a single-hit (Cruz-Chú *et al.*, 2021 ▸). More recently, artificial NN models have become a new avenue for exploring classification solutions with the advent of capable infrastructures (GPUs, machine-learning frameworks) for model training. Shi *et al.* (2019 ▸) uses a CNN for feature extraction and couples its last layer with two additional fully connected (FC) layers that perform binary classification, which achieved an accuracy of 83.8% in predicting single-hits. More recently, another NN-based hit classifier is proposed by Ignatenko *et al.* (2021 ▸). They repurposed YOLO (you only look once) deep-learning models (Redmon *et al.*, 2016 ▸; Redmon & Farhadi, 2018 ▸) from detecting objects to classifying speckle patterns. In fact, these YOLO models also consist of a CNN spatial feature extractor and several FC layers to compress features into the probability of classes and location of objects. However, these models cannot be directly used to classify speckle patterns of previously unseen single-particle samples without example relabeling and model retraining. Their performance with missing detector area is also unknown. YOLO models specifically come with extra complexities, such as requiring bounding boxes as labels and increasing computational cost for finding the location of a speckle pattern.

### 2.2. Similarity metrics in a unified embedding vector space

A common method for assessing the similarity of high-dimensional data, such as images, is to project them onto a unified vector space, often accomplished through feature extraction or embedding. In this unified space, similarity is quantified using metrics like Euclidean distance. One of the early examples was signature verification using a twin NN (Bromley *et al.*, 1993 ▸). During training, a twin NN works on two signatures simultaneously. During verification, only one half of the twin NN is used to map input signatures into a vector space. The output embedding will be compared with previously stored signature embedding in this unified vector space. The stored embedding that is closer to the input embedding is considered to share the same label as the input, thereby, the label of this stored embedding becomes the predicted label of the input. Similar twin NNs were later used to train models for face recognition/verification (Chopra *et al.*, 2005 ▸). Then, triplet NNs (Hoffer & Ailon, 2014 ▸) were applied to further enhance the unified embedding models (Schroff *et al.*, 2015 ▸) by training essentially two twin-NNs on both positive and negative examples simultaneously instead of only one of them. In addition to twin NNs and their variants, many embedding models have been explored for few-shot classification. Vinyals *et al.* (2017 ▸) introduced ‘matching networks’ that map queries and supports to a unified vector space with two independent embedding functions. Snell *et al.* (2017 ▸) proposed that a unified embedding model can be trained with ‘prototypical networks’ so that the embedding of unseen inputs is more likely to be closer to the correct ‘prototype’, defined as the mean of the embedded supports of the same category.

## 3. Methods

**Figure 1:**
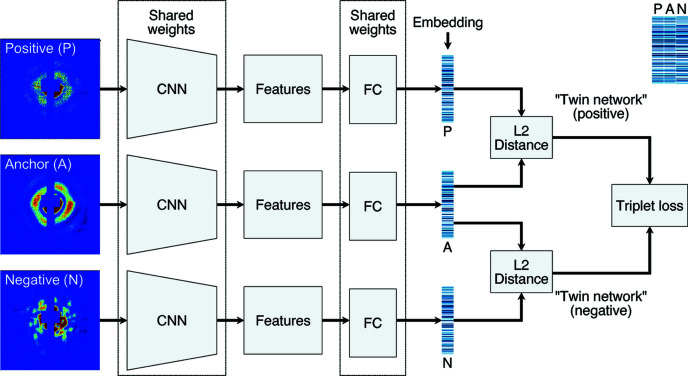
Triplet network architecture for model training. Three input examples (anchor, positive and negative) are propagated through the triplet NN simultaneously. Anchor and positive share the same label, thus forming a matching pair. In contrast, anchor and negative do not share the same label, thus forming an opposing pair. The three CNNs and FC layers share the same weights in the triplet network. After examples are embedded to a low-dimensional vector space, a triplet loss function is used to simultaneously maximize similarities between matching embeddings and minimize those between opposing embeddings. A side-by-side comparison of three embeddings in a triplet are annotated at the upper right corner.

Our speckle pattern classifier uses a unified embedding model to measure pattern similarity through Euclidean distance in the embedding space. This model is trained by two twin NNs simultaneously. One twin NN processes two matching examples that share the same label, while the other works on two opposing examples with different labels. Such dual twin NNs can be further simplified to triplet networks when the matching pair and the opposing pair share a common example. The common example is referred to as an anchor, and the matching example and the opposing example are referred to as positive and negative, respectively. The complete triplet network architecture is summarized in Fig. 1 ▸. In this section, we present the details in model training with triplet networks, including the embedding model, the loss function and the selection of triplet examples. Additionally, we outline the steps for speckle pattern classification.

### 3.1. The embedding model (the vision backbone)

**Figure 2:**
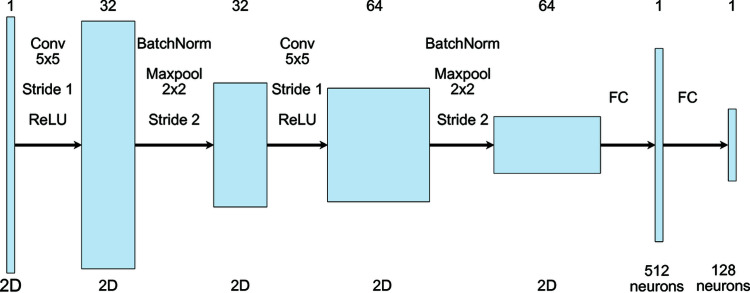
Network architecture of the embedding model. Each shaded rectangle is a volumetric data representation in the NN pipeline. The channel number of each data representation is marked on the top row. The type of spatial dimension, such as two-dimensional tensors or a one-dimensional tensor of neurons, is also annotated for each data representation in the bottom row. Notably, the initial spatial dimension of the input may change if cropping and resizing are applied.

Our embedding model consists of two convolutional layers that extract spatial features and two fully connected layers that compress these features into a low-dimensional vector, or embedding. The detailed architecture of the embedding model is delineated in Fig. 2 ▸. The first convolutional layer uses a 5 × 5 single channel filter with a stride of one and no padding. The second convolutional layer employs a 32-channel 5 × 5 filter with a stride of one and no padding. A ReLU (rectified linear activation unit) activation function is applied to the outcome of each convolutional layer, which is followed by a batch normalization layer and a max-pooling operation performed by a 2 × 2 filter with a stride of two. Then, two fully connected layers are used to generate the final embedding with the size of 128. This way, speckle patterns are encoded into embeddings in a low-dimensional vector space, where the similarity of two embeddings can be evaluated by their squared L2 distance.

### 3.2. Triplet loss function

We train the embedding model in each twin NN using a triplet loss function described by Schroff *et al.* (2015 ▸). Each input consists of a triplet of training examples, which are called anchor *x^a^*, positive *x^p^* and negative *x^n^*. The anchor has the same label as positive, but not negative. Together, (*x^a^*, *x^p^*) forms a matching pair, while (*x^a^*, *x^n^*) forms an opposing pair. During training, three embedding models (CNN + FC) *f* with shared weights map each element in a triplet (*x^a^*, *x_p_*, *x^n^*) into a unified embedding vector space. The objective of training is to separate the two embeddings in each opposing pair by at least a margin of α from the embeddings in the corresponding matching pair in the vector space. Given *N* triplets, the training objective can be stated as 

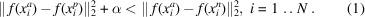

Meanwhile, we enforce that every embedding has a unit length of one in a *d*-dimensional vector space, namely 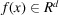 and |*f*(*x*)|_2_ = 1. This means that any speckle pattern will be mapped to a single point on a *d*-dimensional hypersphere with a radius of one. The largest possible value of α is 4 in the squared L2 norm sense.

To facilitate the training, the objective in equation (1) becomes the triplet loss function in equation (2):

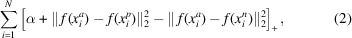

where 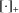 returns zero unless the input value is positive.

### 3.3. Selection of semi-hard triplets

**Figure 3:**
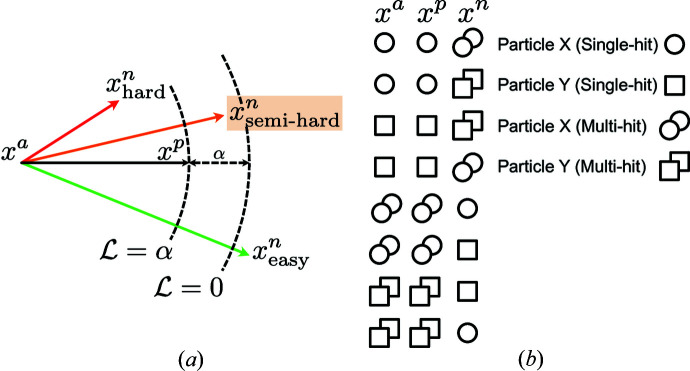
An illustration of the three types of negative examples. (*a*) *x^a^* represents an anchor example and *x^p^* is a positive example. Two arcs in dashed lines, both centered at *x^a^*, are used to divide the embedding space into three areas. The inner arc has a radius of 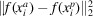 , whereas the outer arc has a radius that is larger by a margin of α. Negative examples will possess three difficulty levels in model training based on the area where they are situated. It is considered a hard negative example if it is located within the inner arc, where 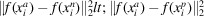 . On the contrary, it is considered an easy negative example when it goes outside the outer arc, where 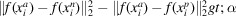 . Lastly, it becomes a semi-hard negative example when it resides in the area bound between the two arcs. Moreover, the loss function results in 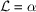 and 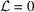 when 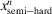 is on the inner arc and outer arc, respectively. Our model training will pull 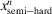 close to the outer arc as much as possible, namely minimizing the loss. (*b*) Illustration of possible semi-hard scenarios when two unique single-particle samples are involved.

A triplet can be randomly selected in three steps: (1) randomly choose a class, (2) randomly sample two unique examples from the chosen class, (3) randomly sample one example from any class other than the chosen class. This method, despite being easy to implement, might not deliver fast convergence when there are too many easy triplets. To explain in detail, we consider three kinds of triplets that might exist during model training: easy, semi-hard and hard, as shown in Fig. 3 ▸(*a*). In an easy triplet, the negative example is already separated by at least a margin of α than the positive example. In a hard triplet, the negative example is actually closer to the anchor than the positive example. In a semi-hard triplet, the negative example is farther away from the anchor than the positive example with a margin smaller than α. The problem with easy triplets is that they contribute to zero in the triplet loss, and thus the model weights will be adjusted only according to other triplets, namely semi-hard and hard triplets. The problem with too many hard examples is that they mostly constitute only a small fraction of the whole population. If the optimization prioritizes separating them from their corresponding anchors, the loss function is more likely to become stuck in bad local minima. Therefore, selecting semi-hard triplets for training is important. This strategy does not intend to ignore hard examples. Instead, once a hard example is pulled into the semi-hard zone, optimization can further drive them into the easy zone. This allows the majority of the negative examples to avoid their anchor by a considerable margin of α. From a practical standpoint, the selection of semi-hard triplets is achieved at the mini-batch level, where our model randomly selects a triplet that satisfies the following condition:

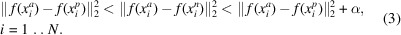

However, complexity arises with semi-hard selections involving multiple single-particle samples. We choose to select random anchor *x^a^* and positive *x^p^* from the same sample with the same label, with the negative *x^n^* selected from any sample with a different label. Under this selection scheme, we lay out all scenarios for semi-hard selections when two unique single-particle samples (particle X and particle Y) are present, as shown in Fig.  ▸(*b*).

### 3.4. Optimization

We trained our NN models using *Adam* (Kingma & Ba, 2017 ▸) with a learning rate of 10^−3^. The model weights are initialized to random values from a Gaussian probability distribution with a mean of 0.0 and a standard deviation of 0.2.

### 3.5. Data augmentation

Data augmentation is widely used in many machine-learning tasks to address limitations imposed by expensive human-labeling and improve model performance. In essence, ‘a data-augmentation is worth a thousand samples’ (Balestriero *et al.*, 2022 ▸). We applied four data augmentation strategies to each speckle pattern in our dataset, including random in-plane rotation, random masking, random zooming, and random shifting in both the horizontal and the vertical directions. Random in-plane rotation mimics the effect of single-particle rotation. Random masking covers some area of a speckle pattern with constant-value pixel intensities to be more robust to bad pixels and parasitic scattering. Random zooming and random shifting enforce the model to learn features independent of detector distance, X-ray wavelength and X-ray beam center. These data augmentation strategies expand the data distribution for the model without manual labeling.

An important caveat when applying data augmentation is to partition the data into a training set and test set before the augmentation. Otherwise it will lead to ‘data leakage’ as explained by Kapoor & Narayanan (2022 ▸). One consequence of ‘data leakage’ is the deceptively good model predictive performance measured on a test set that already contains data augmented or ‘leaked’ from the training set. In other words, the ‘good’ performance can be mostly attributed to model memorization or overfitting rather than generalization.

### 3.6. Four steps in classification

**Figure 4:**
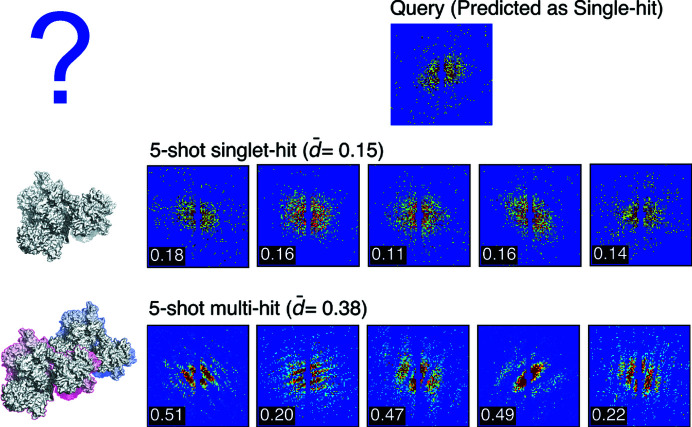
Illustration of a 2-way 5-shot classification. The speckle patterns in this figure are simulated from a type VI secretion system (PDB entry 6n38; Park *et al.*, 2018 ▸). A queried speckle pattern is shown in the first row that has a ground truth label of single-hit. The second and third rows represent the single-hit and multi-hit support sets, respectively. Five patterns are used in each support set. The query-to-support distance is annotated at the bottom left corner of each speckle pattern. The average query-to-support distance is denoted *d* . The single-hit class has a shorter *d* than the multi-hit class, resulting in a single-hit label for the query pattern. Additionally, a question mark in the first row indicates a to-be-determined label of a queried particle. To illustrate a single-hit example, we use a simple cartoon representation of a 6n38 molecule in the second row. Likewise, a multi-hit example is depicted by a cartoon representation of two 6n38 molecules with distinct colored outlines.

Our model maps speckle patterns into a unified embedding space, without directly predicting labels. Instead, label prediction is performed in a query-against-support manner, that is, comparing inputs (queries) to labeled examples (supports). This approach is also referred to as few-shot classification, often implying novel classes for queries and supports. In an *N*-way *X*-shot classification, *N* is the number of classes and *X* is the number of labeled examples per class. The classification takes four steps: (1) embed an unknown input speckle pattern and all support examples in a unified embedding space. (2) Calculate the Euclidean distances from the input to every support example. (3) Average all distances by class. (4) Rank all classes by average distance and select the class with the shortest distance as the label of the unknown speckle pattern. Fig. 4 ▸ demonstrates an example of 2-way 5-shot classification.

## 4. Experiments

The ultimate goal of SpeckleNN is to accurately classify speckle patterns. Our unified embedding model facilitates the conversion of the speckle pattern classification problem into a set of similarity measures. Here we demonstrate two classification solutions, one with offline training and one with online training. Offline training requires past experimental data to train the model, which is then directly applied to classification of speckle patterns in future experiments. However, online training trains the model solely on newly collected data in an ongoing experiment. Both offline and online training are important for SPI experiments in high-data-rate facilities. Offline training offers a ready-to-use model for a wide range of samples, whereas online training provides a potentially more accurate experiment-specific model for the sample of interest.

As mentioned, training an offline model requires speckle patterns from a variety of distinct samples. To date, successful three-dimensional reconstructions from single-particle datasets are primarily from large viruses, such as mimivirus (Seibert *et al.*, 2011 ▸), rice dwarf virus (Hajdu *et al.*, 2016 ▸) and bacteriophage PR772 (Li *et al.*, 2020 ▸). Therefore, we elected to demonstrate the offline-trained model on simulated data. Meanwhile, our model is designed to work on one sample of interest when trained online. We chose to use real bacteriophage PR772 data collected at the LCLS for a demonstration of model training and testing.

### 4.1. Offline training

#### 4.1.1. Dataset

**Figure 5:**
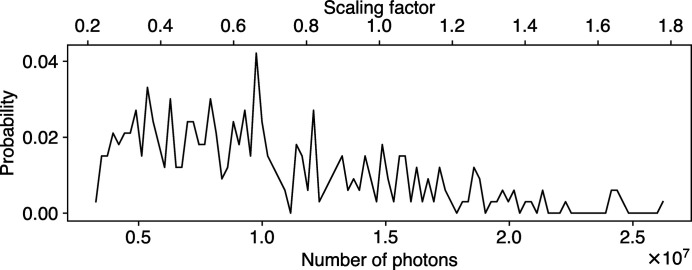
Probability distribution of photon numbers in 332 single-hit speckle patterns, obtained from the LCLS experiment of bacteriophage PR772 at the AMO instrument [experiment ID: amo06156; run numbers: 90, 91, 94, 96 and 102 (Li *et al.*, 2020 ▸)]. Dividing by mean photon numbers (∼1.5 × 10^7^) produces scaling factors specified in the upper *x* axis.

We randomly selected 100 Protein Data Bank (PDB) entries for model training and validation, and another 345 PDB entries for model testing. The number of atoms in those PDB entries ranges from 10^4^ to 10^5^. For every PDB entry, we simulated 400 speckle patterns, with 100 per hit category, from randomly oriented particles in the form of single-hit, double-hit, triple-hit and quadruple-hit. For training purposes, we keep the single-hit label but relabel the rest as multi-hit. The beam profile employed in the simulation has a radius of 0.5 µm and a photon energy of 1.66 keV, and contains 10^12^ photons per pulse. We simulated all speckle patterns using *skopi* (Peck *et al.*, 2022 ▸) on a square detector with the dimensions 172 × 172 pixels. To replicate the conditions similar to real experiments, we first applied a 6 × 8 pixel binary mask mimicking a beam stop at the center and another 172 × 4 pixel binary mask resembling a gap dividing a detector in the middle. Then, X-ray fluence jitter and shot noise were introduced to the dataset. Specifically, during model training, we introduce fluence jitter by rescaling the intensity of speckle patterns with a multiplier sampled from an experimental photon number distribution shown in Fig. 5 ▸. We also added Gaussian noise with zero mean and 0.15 standard deviation. Each speckle pattern is also cropped at the center with a window size of 96 × 96. Data augmentation described in the method section was subsequently applied. Input patterns are intensity normalized prior to model training and inference. Note that the effects of X-ray fluence jitter can not be fully normalized away.

The speckle patterns simulated from the 100 PDB entries are split into 70% for training and 30% for validation. With data augmentation, we obtained 30 000 speckle patterns for model training and another 10 000 for model validation. We found that applying data augmentation can add significant latency to the training process, so decided to cache all speckle patterns into CPU memory. But it is possible to pack even more data for model training through better practices, such as applying data augmentation to a new batch of data while training on the previous batch is still underway.

The test set was formed by simulating speckle patterns from 345 PDB entries. For model prediction, we generated 1000 speckle patterns for each PDB entry through random in-plane rotation as the only data augmentation strategy. The main reason is that speckle patterns are not subject to random masking, random shifting or random zooming as long as the experimental setup remains unchanged. We computed a confusion matrix for each PDB entry and reported accuracy and F-1 scores in the following results.

#### 4.1.2. Performance and photon fluence

**Figure 6:**
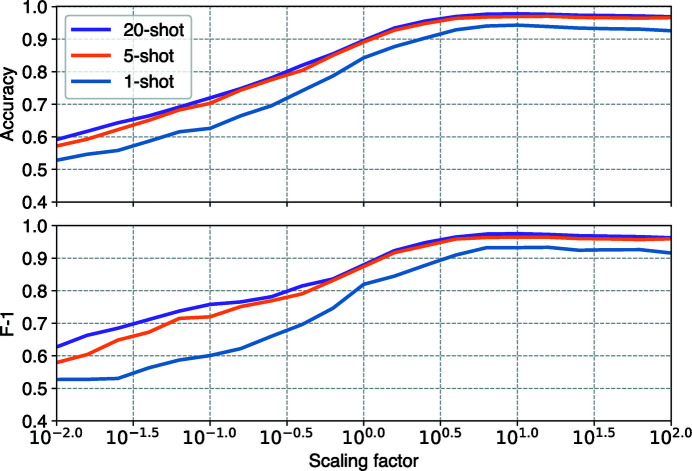
Two-way *X*-shot (*X* = 1, 5, 20) classification performance of our model, as measured by accuracy and F-1 scores under a range of fluence conditions. The baseline fluence is 10^12^ photons per X-ray pulse.

X-ray photon fluence jitter is often present in SPI speckle patterns. To illustrate how fluence jitter affects our model performance, we scanned a range of fluence scaling factors from 10^−2^ to 10^2^ by multiplying by 10^0.5^ at each step. These scaling factors are then applied to simulated speckle patterns in the test set. Meanwhile, we also measured model performance in three unique few-shot classification scenarios, including 1-shot, 5-shot and 20-shot. At the baseline photon fluence, our model achieves an average accuracies of 84.3, 89.1 and 89.6% with 1-shot, 5-shot and 20-shot classifications, respectively. The corresponding F-1 scores are 82.0, 87.4 and 87.9%, respectively. Accuracy and F-1 scores both rise in response to the increase of photon fluence, and converge at about 10^0.6^, ∼4.0× the baseline photon (Fig. 6 ▸).

There are two main lessons we learned from this result. Firstly, the improvement in classification diminishes quickly with increasing support size. For example, a 5-shot classification has a much better performance than a 1-shot classification, but it delivers a comparable performance to a 20-shot classification. If we were to deploy SpeckleNN at an undergoing experiment, it would be more appealing to label only 5 examples per category rather than 1 or 20 examples. Secondly, as free-electron laser technology improves in peak brightness (Li *et al.*, 2022 ▸), the benefit of having higher photon fluence can directly improve the accuracy of SpeckleNN. Interestingly, a 4× photon fluence improvement is sufficient to maximize model performance.

#### 4.1.3. Performance and particle size

**Figure 7:**
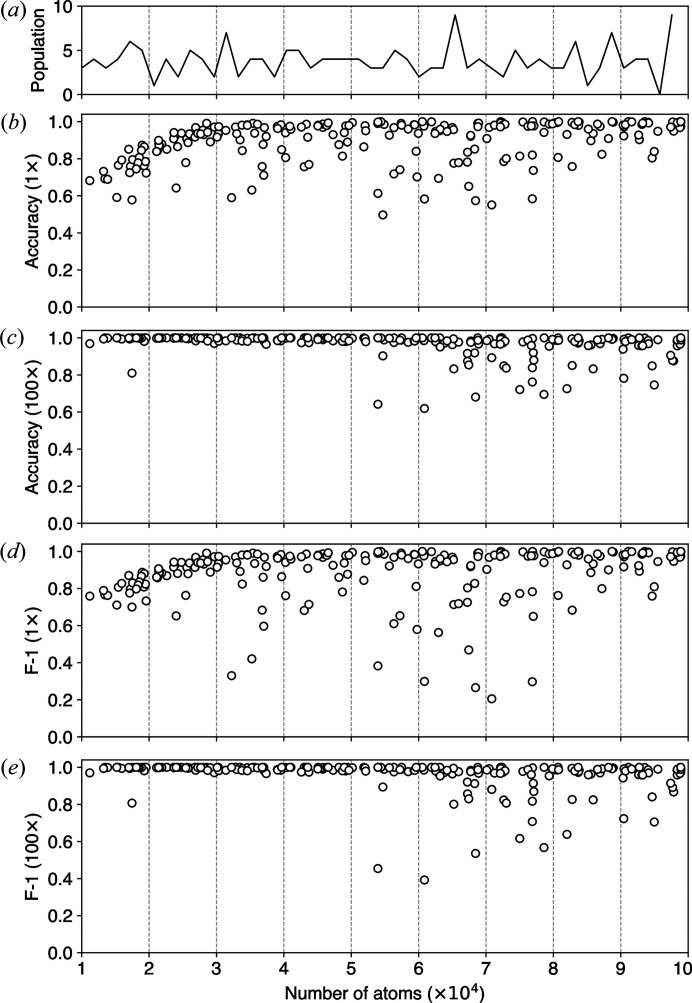
(*a*) Distribution of particle size, as characterized by the number of atoms, among PDB entries used in training the model. (*b*)–(*e*) 2-way 20-shot classification accuracy [(*b*) and (*c*)] and F-1 scores [(*d*) and (*e*)] plotted against the number of atoms in each PDB entry under two respective fluence conditions, 1× the baseline fluence and 100× the baseline fluence. These conditions are labeled on the *y* axes.

PDB entries vary significantly in particle size from hundreds to millions of atoms, and their sizes are unevenly distributed. It is not good practice to form a training dataset by randomly picking PDB entries, which might result in many entries aggregated within a small size range. Models trained on such datasets might perform well only on the highly populated size ranges, but less so otherwise. We pick roughly equal numbers of PDB entries from 20 evenly spaced intervals from 10^4^ to 10^5^ atoms. The distribution of PDB entries over particle size used in our model training is visualized in Fig. 7 ▸(*a*). The PDB entries used in the test set were also sampled from the same size range.

We pointed out that particle size is a limiting factor in model prediction but conditioned on photon fluence. As shown in Fig. 7 ▸(*b*), the average test accuracy of our model at the baseline photon fluence (1× condition) is positively correlated with particle size in the region 1 × 10^4^ to 3 × 10^4^ atoms. The average test accuracy becomes stable when particle size is larger than 3 × 10^4^ despite the presence of outliers. Likewise, we repeated model prediction at 100× larger photon fluence. No further size-dependent correlation is observed across the whole size range, as shown in Fig. 7 ▸(*c*). A similar observation was also evident in the F-1 scores as shown in Fig. 7 ▸(*e*).

**Figure 8:**
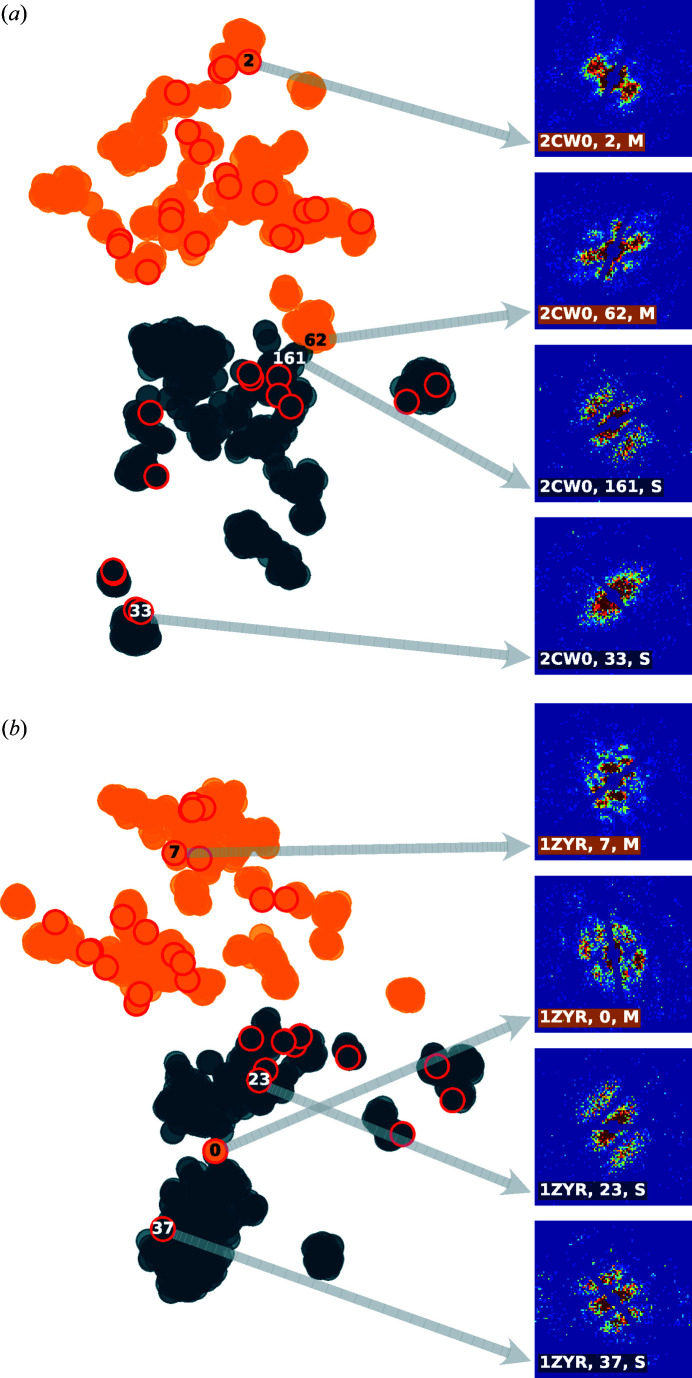
Two-dimensional t-SNE plots depicting the NN embeddings of simulated speckle patterns for PDB entries (*a*) 2cw0 and (*b*) 1zyr (Tuske *et al.*, 2005 ▸). The embeddings of support examples used for *X*-shot classification are highlighted in red. The underlying speckle patterns are visualized on the right side of each t-SNE plot. The numbers provided are solely intended for reference purposes and do not represent any inherent physical significance. In addition, S and M stand for single-hit and multi-hit, respectively.

Despite the overall improvement in average accuracy and F-1 scores resulting from increased photon fluence, our model shows only slight improvement for certain proteins larger than 50 000 atoms. Closer examination revealed that these proteins are pseudo single-hit particles, which are essentially crystallization-induced dimers or higher-order oligomers. In some cases, protein dimers or oligomers can be observed in crystal structures due to the specific packing arrangements of protein molecules within a crystal structure. For instance, Tuske *et al.* (2005 ▸) deposited two *Thermus thermophilus* RNA polymerase holoenzyme structures into the PDB, where 2cw0 is the apo protein and 1zyr is the complex with the antibiotic streptolydigin. Each of the two PDB entries, utilized to simulate speckle patterns, contains two biological units. This led to an interesting situation shown in the t-SNE visualization in Fig. 8 ▸(*a*), where a single-hit speckle pattern, such as speckle No. 161, exhibits pronounced interference patterns similar to those in multi-hit examples like speckle No. 62. In fact, SpeckleNN actually captures the close proximity between these two patterns demonstrated in the t-SNE feature space, indicating its understanding of their similarity. Furthermore, another issue adding to the complexity is the selection of support examples, which are highlighted in red in the same figure. For example, speckle No. 0 in Fig. 8 ▸(*b*), selected as a support example for multi-hit, possesses features that closely align with those pseudo single-hit particles. Unfortunately, the available metadata within these PDB entries do not provide a means to identify these outliers prior to the analysis.

### 4.2. Online training

#### 4.2.1. Dataset

We obtained speckle data from an LCLS experiment of bacteriophage PR772 collected at the AMO instrument [experiment ID amo06156; run numbers: 90, 91, 94, 96 and 102 (Li *et al.*, 2020 ▸)]. We prepared 332 single-hit, 165 multi-hit and 98 non-sample-hit patterns to form our source dataset. Non-sample-hit patterns do not exist in simulated data and are unique to experimental data, which are largely caused by parasitic scattering. We split data into 50% training set, 25% validation set and 25% test set. All speckle patterns were subject to data augmentation, specifically random rotation and random masking, from the source dataset. We limit only 40 and 20 labeled examples per category for model training and validation, respectively. The purpose of imposing this restriction is to reproduce the shortage of labeled examples in a real experiment. However, we applied data augmentation to boost the number of examples per category. Consequently, there were 1374 non-sample-hit, 1288 single-hit and 1338 multi-hit patterns for model training, while there were 1359 non-sample-hit, 1352 single-hit and 1289 multi-hit patterns for model validation. We emphasize that the online model is trained on only the sample used during the experiment and bacteriophage PR772 is the sole specimen used to demonstrate the capabilities of our model. Two-dimensional t-SNE plots of the evolution of the unified embeddings during training are provided in Fig. S1 of the supporting information.

#### 4.2.2. Robust classification despite missing detector area

To illustrate robust classification despite missing detector area, we need to choose a baseline model as a reference point for comparison. We decided to use the model by Shi *et al.* (2019 ▸) for this purpose, which we will refer to as Shi19 herein. It consists of a CNN vision backbone and a multi-layer perceptron (MLP) that outputs probabilities of each label. It reportedly achieved 83.8% accuracy in predicting single-hits. We reimplemented the Shi19 model in *PyTorch* to measure its performance. Note that we need to relabel non-sample-hit and multi-hit as non-single-hit to accommodate the training of the Shi19 model, as it was initially designed for binary classification. We still used the original three labels to train our model, and only relabel them when producing compatible confusion matrices.

A performance comparison between models was conducted for two scenarios: (1) 100% detector area is available, (2) 25% detector area is available. The second scenario is more commonly observed in modular detectors, where a certain area of the detector needs to be masked out due to spurious noise or damaged panels. Sometimes, data from some detector panels must be completely ignored to reduce computation time and thus allow rapid data collection. If speckle pattern classification can be accurately performed on only a fraction of a detector area, it opens the door to solving the ‘data reduction’ problem that bottlenecks high-throughput SPI experiments. That is to say, it can save a considerable amount of time by eliminating the need for assembling and calibrating all detector panels for the classification process.

**Figure 9:**
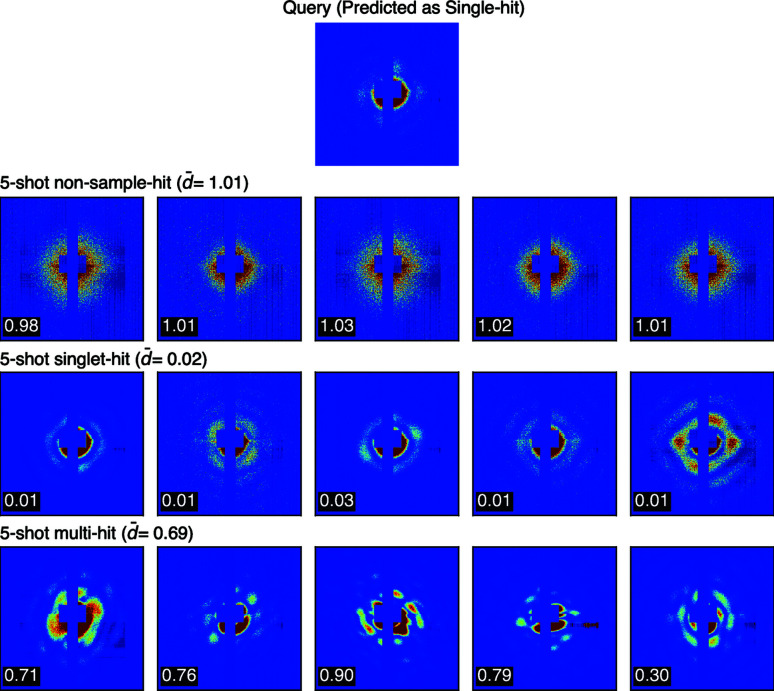
3-way 5-shot classification on speckle patterns with 100% detector area available.

Altogether, we randomly selected 345 single-hit and 655 non-single-hit speckle patterns to form the test set, with non-single-hit made up of 331 non-sample-hit and 324 multi-hit. SpeckleNN classifies speckle patterns in a 5-shot manner, whereas the Shi19 model uses a probability threshold of 0.9 for the classification task. The model accuracy and F-1 scores are summarized in Table 1 ▸. Note that data augmentation enhances the accuracy of the Shi19 model significantly from 83.8 to 98 when 100% detector area is available. Meanwhile, under the same circumstances, SpeckleNN and the Shi19 model have the same accuracy and F-1 scores, respectively. But SpeckleNN outperforms the competing model by a large margin when only 25% detector area is available. Figs. 9 ▸ and 10 ▸ are demonstrations of 3-way 5-shot classification with SpeckleNN on speckle patterns with 100 and 25% detector area available, respectively. This result suggests that SpeckleNN is a more robust speckle pattern classifier and thus better suited for high-throughput SPI experiments. CNN models are also robust to shot-to-shot changes in beam center positions due to the translation invariance property of its convolution operator.

## 5. Conclusions

In this work, we have introduced SpeckleNN, a unified embedding model for real-time speckle pattern classification in X-ray SPI with limited labeled examples. The embedding model, trained with twin NNs, can directly map speckle patterns to a unified vector space, where similarity is characterized by Euclidean distance. We have provided two distinct speckle pattern classification solutions. Firstly, the model trained on multiple samples offline allows few-shot classification of new never-seen single-particle samples. Secondly, the model trained on one sample of interest online exhibits notably improved performance in the presence of substantial missing detector area, compared with the Shi19 model, a simple yet effective NN-based single-particle classifier. The ability of our model to classify speckle patterns with partial detector information presents a significant opportunity for the development of a rapid speckle pattern vetoing process. Additionally, data augmentation is crucial in both offline and online training of our model, with a greater impact in online training.

Although our results show promising progress, future work is needed to transfer the model trained on simulated data to real experimental data. To improve the transferability of the model, two potential approaches can be considered: (1) utilize generative models like diffusion models (Sohl-Dickstein *et al.*, 2015 ▸; Rombach *et al.*, 2021 ▸) or generative adversarial networks (GANs) (Goodfellow *et al.*, 2014 ▸): GANs can be employed to generate more realistic speckle patterns that closely resemble those observed in real world scenarios. By training a generative model to produce synthetic speckle patterns, the model can learn to capture the intricacies and complexities of real data, enhancing the transferability to experimental settings. (2) Employ a mixture of simulation and experimental data: introducing a combination of simulated and experimental data into the training set can facilitate contrastive learning. This approach enables the model to learn the common and distinct characteristics of both datasets, aiding its adaptation to real speckle patterns. 

## Supplementary Material

Supporting figure. DOI: 10.1107/S2052252523006115/if5001sup1.pdf

## Figures and Tables

**Figure 10:**
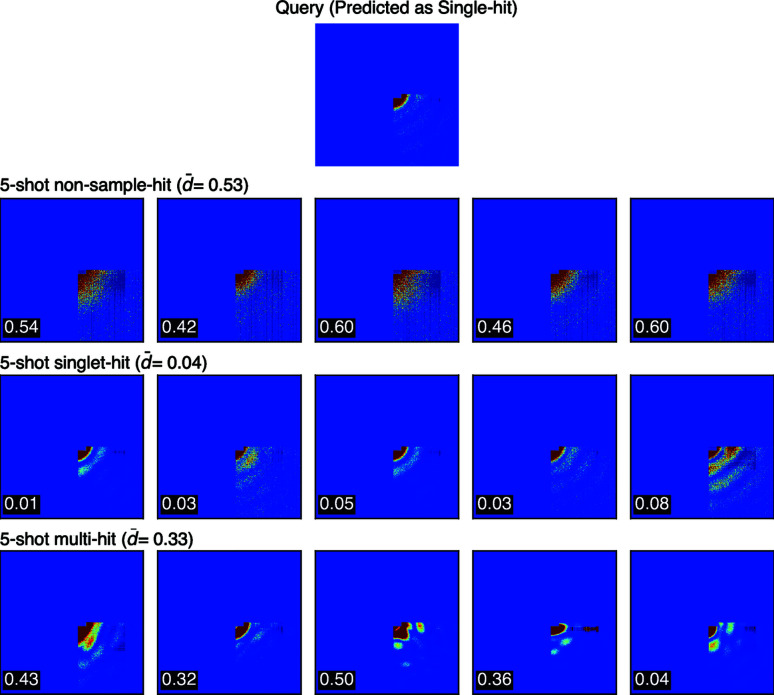
3-way 5-shot classification on speckle patterns with 25% detector area available.

**Table 1:** Model accuracy (Acc) and F-1 scores in two scenarios: (1) 100% detector area is available, (2) 25% detector area is available The percentage detector area visibility is given as a subscript. In addition, we performed a 5-shot classification using SpeckleNN, and the probability threshold used in the Shi19 model is 0.9

Model	Acc_100%_	F-1_100%_	Acc_25%_	F-1_25%_
SpeckleNN	0.98	0.97	0.94	0.92
Shi19 model	0.98	0.97	0.74	0.64
