# Supplementary material for: SpeckleNN: a unified embedding for real-time speckle pattern classification in X-ray single-particle imaging with limited labeled examples
Source: IUCrJ. 2023 Jul 18;10(Pt 5):568–78. doi: 10.1107/S2052252523006115 (PMC10478515; doi:10.1107/S2052252523006115)
Supplement: Supplementary file 1 [file m-10-00568-sup1.pdf]

IUCrJ

Volume 10 (2023)

Supporting information for article:

SpeckleNN: a unified embedding for real-time speckle pattern classification in X-ray single-particle imaging with limited labeled examples

Cong Wang, Eric Florin, Hsing-Yin Chang, Jana Thayer and Chun Hong Yoon

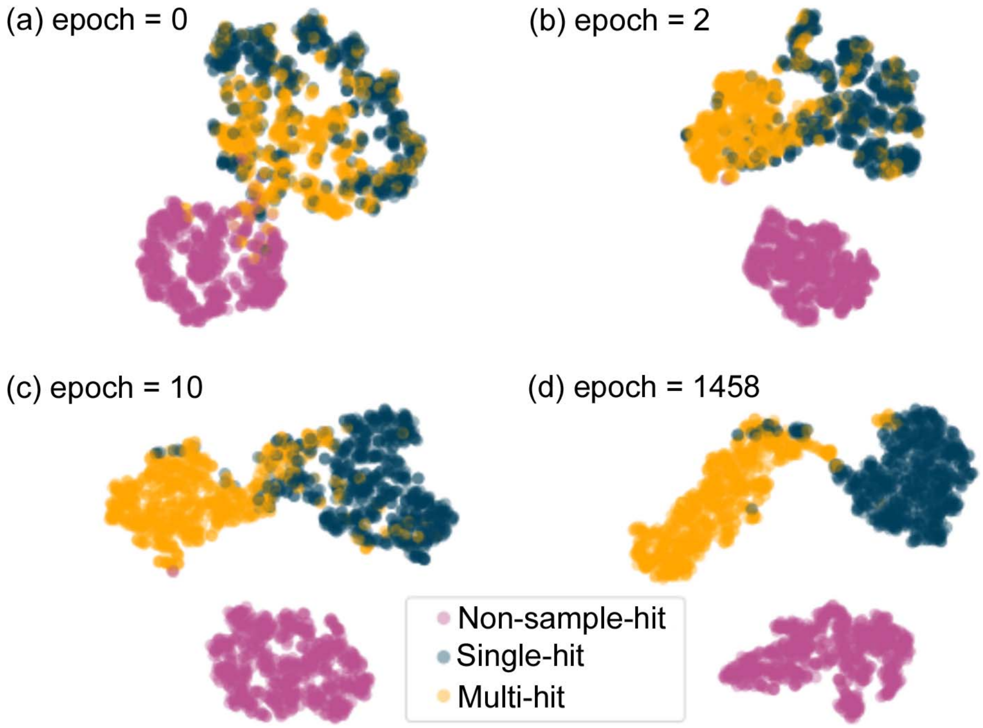

Figure S1. 2D t-SNE plots depicting the neural network embeddings of real speckle patterns of bacteriophage PR772. (a-d) represent four distinct time points (or epochs) during the neural network training process.
